# Temperature-Dependent Fractional Dynamics in Pseudo-Capacitors with Carbon Nanotube Array/Polyaniline Electrodes

**DOI:** 10.3390/nano12050739

**Published:** 2022-02-22

**Authors:** Igor O. Yavtushenko, Marat Yu. Makhmud-Akhunov, Renat T. Sibatov, Evgeny P. Kitsyuk, Vyacheslav V. Svetukhin

**Affiliations:** 1 Laboratory of Diffusion Processes, Ulyanovsk State University, 432017 Ulyanovsk, Russia; yavigor@mail.ru (I.O.Y.); maratmau@mail.ru (M.Y.M.-A.); 2 Scientific-Manufacturing Complex “Technological Centre”, 124498 Moscow, Russia; kitsyuk.e@gmail.com (E.P.K.); v.svetukhin@tcen.ru (V.V.S.)

**Keywords:** pseudocapacitor, carbon nanotube, polyaniline, fractional-order circuit model, memory effect, fractional derivative

## Abstract

Pseudo-capacitors with electrodes based on polyaniline and vertically aligned multiwalled carbon nanotubes (PANI/VA-MWCNT) composite are studied. Fractional differential models of supercapacitors are briefly discussed. The appropriate fractional circuit model for PANI/MWCNT pseudo-capacitors is found to be a linearized version of the recently proposed phase-field diffusion model based on the fractional Cahn–Hilliard equation. The temperature dependencies of the model parameters are determined by means of impedance spectroscopy. The fractional-order α is weakly sensitive to temperature, and the fractional dynamic behavior is related to the pore morphology rather than to thermally activated ion-hopping in PANI/MWCNT composite.

## 1. Introduction

Conductive polymers such as polythiophene, polyaniline (PANI), polyacetylene, etc. have redox properties and can be used as electrode materials for electrochemical power sources. Among these polymers, PANI has proven to be one of the most promising due to its high capacitance characteristics, ease of processing and environmental friendliness [1,2,3,4]. Redox centers in the polymer backbone are not sufficiently stable during many cyclic redox processes [2]. Special additives in composites (activated carbon, nanotubes, graphene, transition metal oxides) are used to eliminate disadvantages of pure PANI such as rapid degradation during cycling and slow ion transfer kinetics. The presence of nanotubes in such composites promotes efficient charge transfer that reduces the internal resistance of the electrodes. Carbon nanotubes (CNTs) increase the electrical conductivity of material regardless of polymer redox state; in addition, a structure with optimal porosity can be created.

For several applications, the stacking of individual CNTs during growth is of great importance. The complex morphology of the entangled nanotube agglomerates leads to a slowdown in the transport of charge carriers [5], including subdiffusive anomalous transport [6]. To eliminate this disadvantageous feature, electrodes based on an array of vertically aligned (oriented) multiwalled carbon nanotubes (VA-MWCNTs) are used [5,7]. This geometry contributes to an increase in the electronic and ionic conductivity in the composite, and the specific active surface area can be larger than in the case of an entangled CNT network.

In this paper, PANI/VA-MWCNT pseudo-capacitors are prepared and the temperature-dependent charging–discharging dynamics of these devices is studied. As is known, fractional-order technique using fractional calculus and fractional equivalent circuits [3] is effective to describe the dynamics of supercapacitors [6,8,9,10,11,12,13,14,15]. Recently, the time-fractional phase-field model has been applied to describe PANI/MWCNT pseudo-capacitors [16]. Here, the simplified representation of a fractional circuit model is used to describe the impedance spectra, cyclic voltammograms, and charging–discharging in potentiostatic mode. The proposed model is a linearized version of the nonlinear model based on the fractional Cahn–Hilliard equation of phase-field diffusion and suitable for the analysis of temperature-dependent fractional dynamics.

Recently, Kopka [17] studied the effect of temperature on the derivative order in the fractional model of supercapacitor. The rate of electrochemical reactions is related to the temperature of the supercapacitor, so the order of fractional derivative model should be temperature-dependent. Here, we determine the temperature dependencies of the fractional model parameters for PANI/VA-MWCNT pseudo-capacitors.

## 2. Materials and Experimental Methods

**Figure 1:**
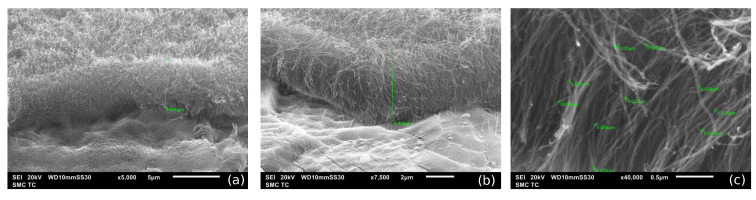
SEM images of a grown MWCNT array on a titanium plate used by us for preparation of PANI/VA-MWCNT pseudocapacitor. Scale bar: 5 μm (**a**), 2 μm (**b**) and 0.5 μm (**c**).

**Figure 2:**
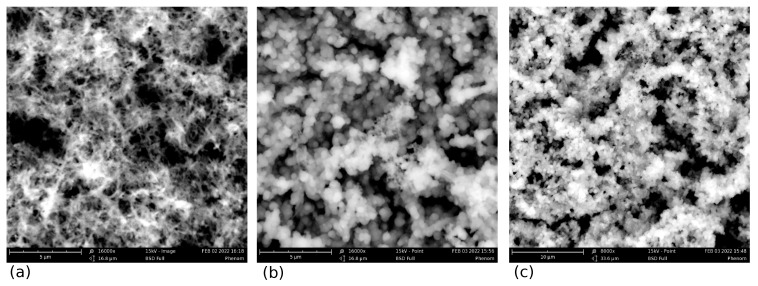
SEM images (top plan view) of the MWCNT array (**a**) and the array covered by single PANI layer (**b**,**c**). Scale bar: 5 μm (**a**), 5 μm (**b**) and 10 μm (**c**).

Pseudo-capacitors with electrodes based on the PANI/VA-MWCNT nanocomposite were prepared. The nanotubes are presented in the form of a vertically aligned array (VA-MWCNT) grown on a 0.5 cm^2^ titanium substrate. The fabrication process starts with wet cleaning and thermal oxidation of a bare silicon base plate to isolate the substrate from the electrodes. Then, Ti and Ni layers were evaporated onto the substrate with the magnetron sputtering system. Ti serves as the current collector material, and Ni particles are the catalyst for CNT forest growth. After the preparation of the VA-MWCNT array shown in Figure 1, the CNT forest was covered with a thin layer of PANI (emeraldine form), obtained by the chemical method of aniline solution oxidation. SEM images (top plan view) of the MWCNT array and the array covered by PANI layers are presented in Figure 2. A two-stage method of coating by polyaniline was used. After drying the first layer, the second layer was applied. The thickness of each layer is approximately equal to 150 nm, and was determined by the method of atomic force microscopy. It is known [1] that the formation of a PANI layer on the CNT surface begins with the adsorption of aniline molecules, which then form oligomers during oxidative polymerization. When such a mechanism is implemented, the properties of the resulting PANI are significantly affected by the nature of the surface groups of the initial CNTs.

**Figure 3:**
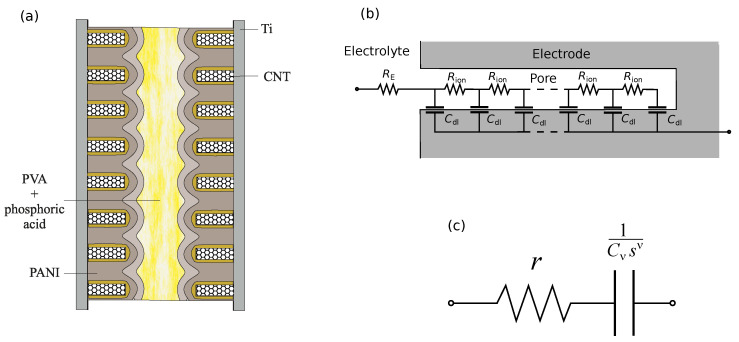
Schematic representation of a cell with electrodes based on vertically aligned MWCNT array/PANI composite (**a**), RC transmission line (De Levie model) for a single pore (**b**), and the simplest equivalent circuit model of a supercapacitor (**c**).

The electrolyte in our system is a solution of phosphoric acid H${}_{3}$PO${}_{4}$ and polyvinyl alcohol (PVA). A schematic representation of the PANI/VA-MWCNT pseudocapacitor is shown in Figure 3a.

We determine the model parameters of pseudo-capacitors by fitting impedance spectra, cyclic voltammograms and charging–discharging curves. These data were obtained by measurements with a P-45X potentiostat–galvanostat (Electrochemical Instruments company). For cyclic voltammetry, the voltage ranges from −0.5 to 0.5 V, potential scan rates are 20, 50 and 100 mV/s. For the impedance spectroscopy measurement, the frequency ranges from 0.1 Hz to 50 kHz, and the voltage amplitude is 50 mV.

## 3. Fractional Differential Models of Supercapacitors

It is common to analyze impedance results using a physical model that is expressed by a system of mathematical equations. If this system is linear, it can be usually represented by an equivalent circuit. The parameters of circuit electrical components are related to the physical and chemical properties of electrolyte, electrode and their interface. The charging–discharging kinetics of supercapacitors and pseudo-capacitors is largely determined by the diffusion of ions in electrodes and electrolyte.

The de Levie model [18] successfully describes the impedance of a porous electrode containing oblong pores (see, e.g., [19,20]). The impedance of a single pore is modeled by a transmission line (Figure 3b) with the assumption that specific resistances of solution and local impedance do not depend on the depth inside pore, and the solid phase is assumed to be perfectly conducting [18]. The half-integer impedance $Z=\sqrt{R/j\omega C}$ characterizes this transmission line. A more general form is given by a constant phase element (CPE),

(1) $$Z\left(\omega \right)=\frac{1}{C_{\nu }{\left(j\omega \right)}^{\nu }}.$$

CPE coupled in series with resistor *r* (Figure 3c) represents a simplified supercapacitor model considered in references [14,21],

(2) $$Z\left(s\right)=R+\frac{1}{C_{\nu }s^{\nu }},\;s=j\omega ,$$

and used in [22,23] to characterize electric double layer (EDL) supercapacitor impedance. Here, *s* can be associated with the Laplace variable. In [24], this impedance model is used to predict the transient response of a supercapacitor to a voltage-step signal. The de Levie model successfully describes electrodes with pores of similar geometric parameters, particularly nanocrystalline TiO${}_{2}$ films [25], and other metal oxides electrodes. Transition metal oxides such as TiO${}_{2}$, NiO${}_{x}$, RuO${}_{2}$, MnO${}_{x}$ are widely used in the development of supercapacitor electrodes [26,27,28]. It is noteworthy that the hierarchical structure of the electrode surface also leads to a fractional impedance, which can be derived within the recursive fractal ladder model [29].

In review [24], the authors discuss three equivalent circuit models for EDL supercapacitors. The first of them is shown in Figure 3c. Another model is a combination of a resistor and three CPEs. In this case, the impedance is

(3) $$Z\left(s\right)=R+\frac{1}{C_{\alpha }s^{\alpha }}+\frac{1}{C_{\beta }s^{\beta }}+\frac{1}{C_{3}s^{\alpha +\beta }}.$$

This model was used in [10] to describe the dynamics of supercapacitor HE0120C-0027A 120 F in the frequency range 1 mHz–1 kHz. The third circuit model is given by impedance

(4) $$Z\left(s\right)=R+k\frac{{\left(1+s/\omega_{0}\right)}^{\alpha }}{s^{\beta }}.$$

This was proposed in [23] and successfully applied to the description of supercapacitor EPCOS 5 F. The fitted parameters are as follows α=0.5190, β=0.9765, k=0.3440 Ω/s${}^{\beta }$.

For the above-listed impedance models, the corresponding charging–discharging equations for current and voltage contain fractional derivatives. On the other hand, due to heterogeneity and complexity of porous electrodes, anomalous diffusive kinetics of ions can take place [13,14,15]. Anomalous diffusion is characterized by power law expansion of the diffusion packet, $\Delta \left(t\right)\propto t^{\alpha /2}$, with α≠1. The case 0<α<1 is classified as subdiffusion, and the case α>1 as superdiffusion. Mathematical treatment of self-similar anomalous diffusion is usually based on diffusion equations with fractional derivatives.

The simplest fractional diffusion equation has the form

(5) $$\frac{\partial c(x,t)}{\partial t}=K{\;}_{0}D_{t}^{1-\nu }\frac{\partial^{2}c\left(x,t\right)}{\partial x^{2}},$$

where

$${}_{0}D_{t}^{1-\nu }c\left(x,t\right)=\frac{1}{\Gamma (\nu )}\frac{\partial }{\partial t}\int_{0}^{t}\frac{c(x,\tau )}{{\left(t-\tau \right)}^{1-\nu }}d\tau ,\;0<\nu \leq 1,$$

is the fractional Riemann–Liouville derivative of order 1−ν [30].

Using anomalous diffusion equations with fractional derivatives, one could generalize impedances for different geometries and boundary conditions (see [25,31] and references therein). The simplest example is subdiffusive generalization of Warburg’s impedance for a semi-infinite medium [31]

(6) $$Z\left(j\omega \right)=B{\left(i\omega \right)}^{-(1-\nu /2)},$$

where *B* is a frequency-independent constant.

### 3.1. Havriliak–Negami Response

To evaluate electrolyte diffusion parameters in porous media, electrochemical impedance spectroscopy is often used. In [18,32,33], the relationship between pore size and electrochemical properties of electrodes has been studied. One of the approaches to assessing the properties of a supercapacitor from impedance spectra is based on the formal representation of a supercapacitor as a dielectric liquid in which the molecular relaxation of the system is assessed in a wide range of frequencies and associated with its structure. Such a view is convenient for using widely known models of dielectric relaxation, such as the Debye, Cole–Cole, and Havriliak–Negami (HN) models. Unlike capacitance, resistance and leakage current, dielectric permeability is an intensive rather than extensive characteristic of the system. Studies [34,35] have shown that structural confinement has a significant effect on molecular relaxation, and it is possible to estimate the contribution of surface morphology by the electrolytic molecular component [34].

The circuit element based on the HN function describes the asymmetric and broad nature of dielectric dispersion [36]. In the case of a linear response, the relationship between current and voltage can be represented as follows

$$i\left(t\right)=K\frac{d}{dt}\int_{0}^{\infty }\phi \left(t^{\prime }\right)\;u\left(t-t^{\prime }\right)dt^{\prime }.$$

Turning to the Fourier transforms, we obtain

$$\frac{\tilde{i}\left(j\omega \right)}{\tilde{\phi }\left(j\omega \right)}=K\cdot j\omega \cdot \tilde{u}\left(j\omega \right).$$

In the case of a system with the HN response, we have

$${\left[1+{\left(j\omega \tau \right)}^{\alpha }\right]}^{\beta }\;\tilde{i}\left(j\omega \right)=K\cdot j\omega \cdot \tilde{u}\left(j\omega \right).$$

The inverse Fourier transformation leads to a fractional differential relationship

$${\left[1+\tau^{\alpha }{\;}_{-\infty }D_{t}^{\alpha }\right]}^{\beta }\;i\left(t\right)=g\left(t\right),\;g\left(t\right)=K\dot{V}\left(t\right)$$

In the case of a step input $V\left(t\right)=V_{0}l\left(t\right)$, we have

$${\left[1+\tau^{\alpha }{\;}_{-\infty }D_{t}^{\alpha }\right]}^{\beta }\;i\left(t\right)=KV_{0}\delta \left(t\right).$$

Here, l(t) is the Heaviside step function.

The solution of this equation is expressed through the generalized Mittag–Leffler function proposed by Prabhakar [37],

$$E_{\alpha ,\gamma }^{\beta }\left(z\right)=\sum_{n=0}^{\infty }\frac{\Gamma (\beta +n)}{\Gamma (\beta )\Gamma (\alpha n+\gamma )n!}z^{n}.$$

Using the Laplace transform

$$\int_{0}^{\infty }e^{-st}\;t^{\gamma -1}E_{\alpha ,\gamma }^{\beta }\left(at^{\alpha }\right)dt=\frac{s^{\alpha \beta -\gamma }}{{\left(s^{\alpha }-a\right)}^{\beta }},$$

one can express the relaxation function in the form

$$f\left(t\right)=i\left(t\right)/KV_{0}=\tau^{-\alpha \beta }t^{\alpha \beta -1}E_{\alpha ,\alpha \beta }^{\beta }\left(-{\left(t/\tau \right)}^{\alpha }\frac{}{}\right).$$

The asymptotic behavior of the solution at large and small times is given by power laws

$$f\left(t\right)∼\Gamma \left(1-\alpha \beta \right)\;\tau^{-\alpha \beta }t^{-1+\alpha \beta },\;t\to 0,$$

$$f\left(t\right)∼\alpha \beta \tau^{\alpha }{\left[\Gamma \left(1-\alpha \right)\right]}^{-1}\;t^{-1-\alpha },\;t\to \infty .$$

If β=0, response f(t) is expressed through the two-parameter Mittag-Leffler function.

The HN response is often considered to be a general expression for the universal relaxation law [38]. This universality implies the similarity of relaxation laws in different materials. This universality holds for dielectric relaxation in dipolar and nonpolar materials, for hopping transport in semiconductors, conduction in ionic materials, delayed luminescence decay, surface conduction on insulators, kinetics of chemical reactions, mechanical relaxation, magnetic relaxation. Despite the completely different internal mechanisms, the processes show striking similarity [38]. This universality stimulates the search for an appropriate stochastic model for the universal relaxation law. Investigations of such kind have been carried out in many works (see e.g., [39,40,41,42]). Based on the solution of fractional relaxation equation [43] and HN response [44], the memory recovery effect was demonstrated. Corresponding relaxation curves are described by the exponential law at initial stage and power law for long-time asymptotics. Charging–discharging curves in PANI/VA-MWCNT demonstrate the similar behavior (see Section 3.3).

### 3.2. Phase-Field Model

In [16], a generalized diffusion impedance model for materials with a subdiffusion phase transition is proposed. The model is based on the fractional Cahn–Hilliard equation with fractional time derivatives. A one-dimensional cell with reflecting and absorbing boundaries is considered. Phase-field generalizations of anomalous diffusion models AD-Ib and AD-Ia presented in [31] are described by the following time-fractional equations [16] with Caputo and Riemann–Liouville derivatives:

$${}_{0}^{C}D_{t}^{\alpha }c=M\;\nabla \left(c\;\nabla \mu_{a}\right),{\;}_{0}^{RL}D_{t}^{\alpha }c=M\;\nabla \left(c\;\nabla \mu_{a}\right).$$

Here, *M* is the ambipolar mobility, and $\mu_{a}$ is the effective (ambipolar) chemical potential (see details in [16]).

The corresponding impedance models were denoted as $Z^{C-\mathrm{CH}}$ and $Z^{\mathrm{RL}-\mathrm{CH}}$, respectively. Letters denote the type of used fractional time derivative (Caputo or Riemann–Liouville):

(7) $$Z^{C-\mathrm{CH}}\propto \frac{\sqrt{\Lambda (\omega )}}{i\omega \;}F\left(\omega \right),\;Z^{\mathrm{RL}-\mathrm{CH}}\propto \frac{1}{\sqrt{\Lambda (\omega )}}F\left(\omega \right)$$

with

$$\Lambda \left(\omega \right)={\left(i\omega \right)}^{\alpha }.$$

The form of F(ω) depends on boundary conditions. For a cell with reflecting boundary, in [16], it was obtained

$$F_{\mathrm{refl}}\left(\omega \right)=\frac{{\left(1+\sqrt{1-4\chi \Lambda (\omega )}\right)}^{3/2}\coth\left[\frac{l}{\sqrt{2\chi }}\sqrt{1-\sqrt{1-4\chi \Lambda (\omega )}}\right]-{\left(1-\sqrt{1-4\chi \Lambda (\omega )}\right)}^{3/2}\coth\left[\frac{l}{\sqrt{2\chi }}\sqrt{1+\sqrt{1-4\chi \Lambda (\omega )}}\right]}{\sqrt{1-4\chi \Lambda (\omega )}}.$$

**Figure 4:**
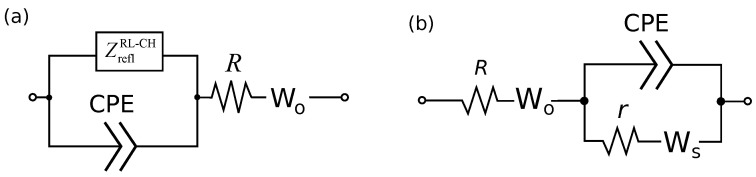
Equivalent circuit model of PANI-MWCNT pseudocapacitor with fractional phase-field element (**a**), and simplified model (**b**).

The frequency dependencies of the PANI/VA-MWCNT pseudocapacitor impedance were described by an equivalent circuit (Figure 4a) containing generalized fractional elements $Z_{\mathrm{refl}}^{C-\mathrm{CH}}$ or $Z_{\mathrm{refl}}^{\mathrm{RL}-\mathrm{CH}}$ defined by (7).

The proposed equivalent circuit was substantiated by the following arguments [16]. The ion transport is interpreted in terms of the one-dimensional diffusion model. The schematic representation of the pseudocapacitor is given in Figure 3a. According to the de Levie model [18,19], CPE describes the EDL capacity formed around the MWCNTs. Diffusion of ions in the interelectrode space is described by the open Warburg impedance. Generalized fractional element $Z_{\mathrm{refl}}^{\mathrm{RL}-\mathrm{CH}}$ corresponds to phase-field ion diffusion in PANI filling the VA-MWCNT array. Reflecting boundary condition is assumed for the base of nanotube array. The RL-CH model (Riemann–Liouville type) implies non-conserving ion density, and it is related to the EDL formation by fraction of ions during phase-field diffusion in PANI filling the MWCNT forest. The series resistor corresponds to the summarized resistance of MWCNTs, polymer and electrolyte.

### 3.3. Linearized Model

The model described in the previous section implies phase-field diffusion of ions in PANI filling the MWCNT forest. The Cahn–Hilliard equation and the corresponding circuit model are nonlinear [16]. The expressions for impedance are obtained after linearization (for details, see [16]). The equivalent scheme is dependent on state of charge. For simplicity, under small voltage perturbations phase-field diffusion can be replaced by ordinary diffusion (Figure 4). Such a replacement implies the dependence of diffusion coefficient on the reference values of ion concentration. Below, we will show that this simplified model describes the observed impedance spectra of PANI/VA-MWNT pseudo-capacitors quite well.

**Figure 5:**
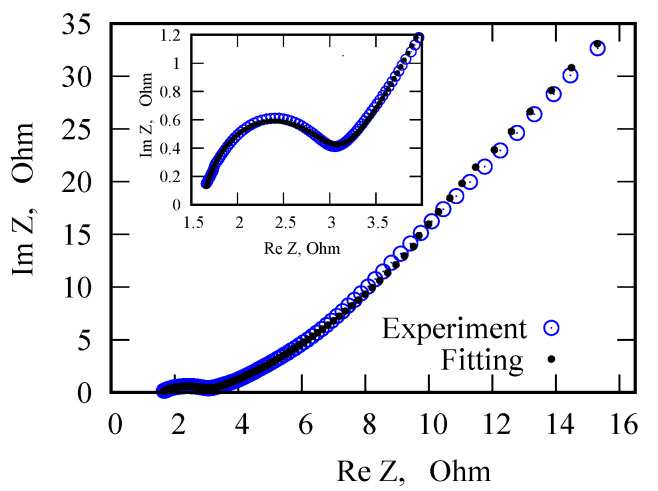
Nyquist plot for PANI/VA-MWCNT pseudo-capacitor.

**Figure 6:**
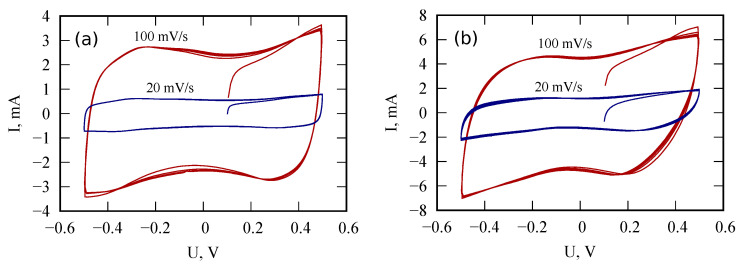
Cyclic voltammograms of PANI/VA-MWNT supercapacitors with one (**a**) and two (**b**) PANI layers. Scan rates are 20 and 100 mV/s.

To study the effect of PANI layer thickness on the characteristics of PANI/VA-MWCNT pseudocapacitor, samples with one and two PANI layers were studied. The fitted parameters for impedance spectra are provided in Table 1. The used equivalent circuit is shown in Figure 4b. A comparison of the model impedance spectra with the measured ones is presented in Figure 5. Cyclic voltammograms of PANI/VA-MWNT pseudo-capacitors with single and double PANI layers demonstrated in Figure 6 indicate that the sample with double PANI layer is characterized by higher capacity (0.05 F) than the single layer pseudo-capacitor (0.025 F).

CPE is characterized by impedance $Z_{\mathrm{CPE}}=C_{\alpha }^{-1}{\left(j\omega \right)}^{-\alpha }$. Two Warburg elements ($W_{s}$ and $W_{o}$) are included into the circuit. Element $W_{s}$ corresponds to the one-dimensional diffusion in a finite cell with an absorbing boundary, the $W_{o}$ element is the same with a reflecting boundary. The corresponding impedances are

$$Z_{W_{s}}=\frac{W_{s}}{\sqrt{j\omega }}\tanh\left(b_{s}\sqrt{j\omega }\right),\;Z_{W_{o}}=\frac{W_{o}}{\sqrt{j\omega }}\coth\left(b_{o}\sqrt{j\omega }\right),$$

where $W_{s}$ and $W_{o}$ are Warburg coefficients, $b_{s,o}=d/\sqrt{D}$, where *d* is thickness of the Nernst diffusion layer, *D* is the diffusion coefficient.

The EIS Spectrum Analyzer software is used to fit the impedance spectroscopy data. The Levenberg–Marquard algorithm with amplitude minimization has been chosen.

**Figure 7:**
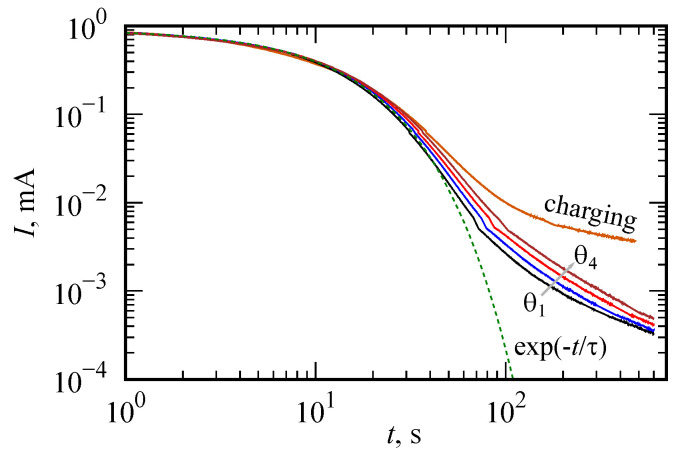
Charging and discharging curves |I(t)| in the log–log scale. Charging time θ is varied: θ=30, 60, 120, 240 s. The initial stage is successfully approximated by an exponential function with a relaxation time τ=12 s.

Figure 7 demonstrates the charging current curve and discharging curves for different charging times (θ=30, 60, 120, 240 s). A slight jump noticeable on the curves is associated with a change in the measuring range of the device. The initial stage is successfully approximated by an exponential function with a relaxation time τ=12 s. Long-term relaxation is dependent on prehistory of charging process. This is a sign of nonlocality in time behavior that is consistent with the fractional circuit model discussed in this work.

## 4. Temperature-Dependent Fractional Dynamics in PANI/VA-MWCNT Pseudo-Capacitors

The rate of electrochemical reactions is related to the temperature of the supercapacitor, so the order of fractional derivative model should be temperature-dependent [17]. Here, we determine the temperature dependencies of the fractional model parameters for PANI/MWCNT pseudo-capacitors.

**Figure 8:**
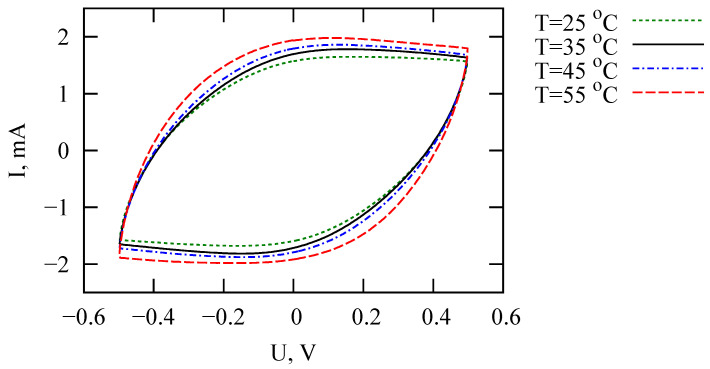
Cyclic voltammograms (10th cycle, scan rate is 50 mV/s) of PANI/VA-MWCNT pseudo-capacitors for different temperatures.

The proposed fractional circuit is consistent with the results of measurements by cyclic voltammetry, impedance spectroscopy and charge–discharge in potentiostatic mode. We made sure that the proposed model works satisfactorily for different temperatures. The temperature dependencies of the fractional model parameters are studied. Equivalent circuit model parameters for T=25 ${}^{\circ }$C, T=35 ${}^{\circ }$C, T=45 ${}^{\circ }$C, and T=55 ${}^{\circ }$C are listed in Table 2. Cyclic voltammograms of PANI/VA-MWCNT pseudocapacitor for different temperatures are demonstrated in Figure 8.

**Figure 9:**
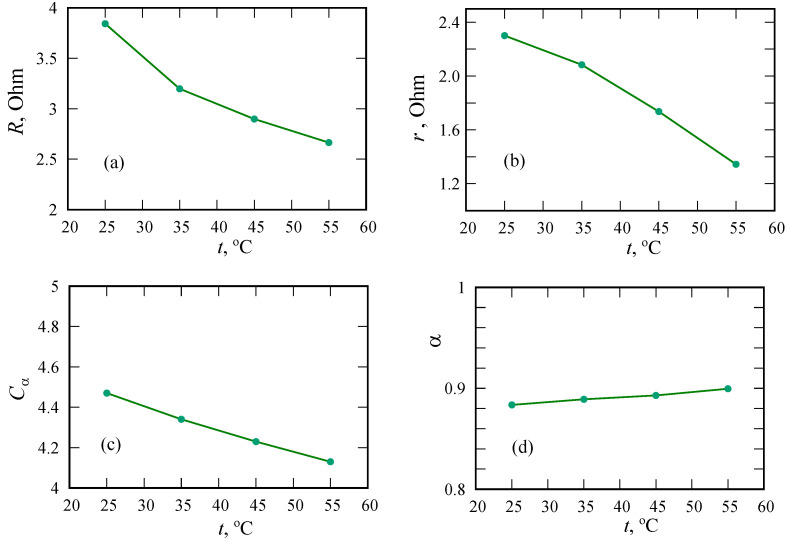
Equivalent circuit model parameters for different temperatures. Resistances *R* (**a**) and *r* (**b**), CPE parameter $C_{\alpha }$ or fractional capacity in $10^{-5}$ s${}^{\alpha }\cdot$Ohm${}^{-1}$ (**c**) and fractional order α (**d**).

The resistances *R* and *r* decrease with increasing temperature (Figure 9). Apparently, both parameters are associated with the transfer of ions in the PANI/MWCNT structure. The α parameter is weakly sensitive to temperature, which means that it is related to the pore morphology. It is expected that in the case of the dominant role of ion-hopping transport in PANI, a significant dependence of α on temperature would be observed. The $C_{\alpha }$ parameter decreases, even though voltammograms indicate a slight increase in the supercapacitor’s capacity with increasing temperature. Changes in the parameters of Warburg elements can be traced from the data in Table 2.

In contrast to the results of reference [17], we observe an increase in the fractional exponent with increasing temperature, which is, in some sense, consistent with the theory of dispersive transport in disordered materials [41,45].

## 5. Conclusions

Pseudo-capacitors with electrodes based on PANI/VA-MWCNT composites have been manufactured and investigated. The measured discharge curves demonstrate the presence of a memory effect in the devices under study, and the impedance spectra are described by a fractional-order equivalent circuit model, which can be justified within the framework of the anomalous diffusion-reaction model or transmission line model. The proposed model is a linearized version of the nonlinear model based on the fractional Cahn–Hilliard equation of phase-field diffusion [16]. The fractional-order equivalent circuit is consistent with the measurements by cyclic voltammetry, impedance spectroscopy, and potentiostatic charging–discharging. We investigate the temperature dependence of the parameters of the fractional model. The resistances *R* and *r* associated with the transfer of ions in the PANI/VA-MWCNT structure decrease with increasing temperature. The fractional-order α is weakly sensitive to temperature. This fact indicates that fractional behavior is related to the pore morphology rather than to thermally activated ion-hopping in the PANI/VA-MWCNT composite. In contrast to the results of reference [17], we observe a weak increase in the fractional exponent with increasing temperature, which is consistent with the dispersive transport theory for disordered materials [46].

## Figures and Tables

**Table 1:** Parameters of the equivalent circuit model.

Parameter	PANI/VA-MWCNT 1	PANI/VA-MWCNT 2
*R*, Ohm	4.124	1.623
*r*, Ohm	0.704	1.360
$C_{\alpha }$, $10^{-4}$ s${}^{\alpha }\cdot$Ohm${}^{-1}$	5.558	1.270
α	0.8068	0.8654
$W_{s}$, Ohm·s${}^{-1/2}$	6.497	10.513
$b_{s}$, s${}^{1/2}$	2.408	2.272
$W_{o}$, Ohm·s${}^{-1/2}$	8.081	0.348
$b_{o}$, s${}^{1/2}$	0.238	0.0263

**Table 2:** Equivalent circuit model parameters for different temperatures.

Parameter	T=25 ${}^{\circ }$C	T=35 ${}^{\circ }$C	T=45 ${}^{\circ }$C	T=55 ${}^{\circ }$C
*R*, Ohm	3.8431	3.1989	2.8983	2.6658
*r*, Ohm	2.301	2.085	1.737	1.345
$C_{\alpha }$, $10^{-5}$ s${}^{\alpha }\cdot$Ohm${}^{-1}$	4.47	4.34	4.23	4.13
α	0.88363	0.88906	0.89299	0.89952
$W_{s}$, Ohm·s${}^{-1/2}$	40.478	39.609	38.988	35.463
$b_{s}$, s${}^{1/2}$	6.7649	18.647	25.284	7.0558
$W_{o}$, Ohm·s${}^{-1/2}$	3.1208	2.3203	0.54176	1.656
$b_{o}$, s${}^{1/2}$	0.0907	0.085134	0.020342	0.066649
